# COVID-19 Vaccine Hesitancy among Pregnant Women Attending Antenatal Clinics in Pakistan: A Multicentric, Prospective, Survey-Based Study

**DOI:** 10.3390/v14112344

**Published:** 2022-10-25

**Authors:** Zia Ul Mustafa, Shazma Bashir, Arfah Shahid, Iqra Raees, Muhammad Salman, Hamid A. Merchant, Mamoon A. Aldeyab, Chia Siang Kow, Syed Shahzad Hasan

**Affiliations:** 1 Discipline of Clinical Pharmacy, School of Pharmaceutical Sciences, Universiti Sains Malaysia, Penang 11800, Malaysia; 2 Department of Pharmacy Services, District Headquarters (DHQ) Hospital, Pakpattan 57400, Pakistan; 3 School of Health, Sport and Bioscience, University of East London, Stratford Campus, London W1S 3PR, UK; 4 Department of Medicine, Rawalpindi Medical University, Rawalpindi 46000, Pakistan; 5 Department of Medicine, Faisalabad Medical University, Faisalabad 38000, Pakistan; 6 Institute of Pharmacy, Faculty of Pharmaceutical and Allied Health Sciences, Lahore College for Women University, Lahore 54000, Pakistan; 7 Department of Pharmacy, School of Applied Sciences, University of Huddersfield, Huddersfield HD1 3DH, UK; 8 Department of Pharmacy Practice, School of Pharmacy, International Medical University, Kuala Lumpur 57000, Malaysia

**Keywords:** vaccine uptake, SARS-CoV-2, pregnancy, gestation, South Asia, Muslim

## Abstract

This study aimed to assess the vaccination status and factors contributing to vaccine hesitancy among pregnant women in the largest province of Pakistan. A multicentric, prospective, survey-based study using an interviewer-administered tool was conducted among pregnant women attending antenatal clinics between 1 December 2021 through 30 January 2022 across seven hospitals in Pakistan. The healthcare professionals providing care at the participating hospitals administered the survey. Four hundred and five pregnant women fully consented and completed the study. The majority of the study participants (70.6%, n = 286) were aged between 25 and 34 and had a previous successful pregnancy history. More than half of the study participants (56.0%, n = 227) did not receive COVID-19 vaccination at the time of data collection despite their family members (93.9%, n = 372) had already received at least one dose of COVID-19 vaccine. Among those who received COVID-19 vaccination (n = 173), vaccine efficacy, protection for the foetus, and risk of COVID-19-associated hospitalisation were the main driving factors for vaccine hesitancy. The majority of the unvaccinated women (77.8%, n = 182) had no intention of receiving the vaccine. However, more than two-thirds (85.7%, n = 342) consulted the doctor about COVID-19 vaccines, and most were recommended to receive COVID-19 vaccines by the doctors (80.7%, n = 280). Women were significantly more likely to be vaccinated if they had employment (odds ratio [OR] 4.47, 95% confidence interval [CI]: 2.31–8.64) compared with their counterparts who were homemakers, consulted their doctors (OR 0.12, 95% CI: 0.04–0.35), and if they did not have pregnancy-related issues (OR 6.02, 95% CI: 2.36–15.33). In this study, vaccine hesitancy was prevalent, and vaccine uptake was low among pregnant women. Education and employment did impact COVID vaccination uptake, emphasising the need for more targeted efforts to enhance the trust in vaccines.

## 1. Introduction

Almost two years into the COVID-19 pandemic, this deadly disease seems to be still gripping many countries, with emerging variants of concern setting off alarm bells globally [1]. In addition, recent data indicate an association between COVID-19 history and adverse maternal and neonatal outcomes [2,3,4,5]. Thus, it has become evident that high vaccination rates are paramount to establishing herd immunity worldwide, the most effective strategy to combat the pandemic [6]. Consequently, multiple vaccine platforms are being used, ranging from conventional formulations, including live attenuated, inactivated, protein subunit, and virus-like particles (VLPs), to emerging novel technologies such as nucleic acid (mRNA) and viral vector-based vaccines [7].

Cardiopulmonary and immunological adaptations during pregnancy predispose women to an increased risk of developing severe COVID-19, including admission to the intensive care unit (ICU) and respiratory failure, as well as potential pregnancy complications such as preterm birth and caesarean delivery. Worse still, cases of vertical transmission have also been reported [8,9,10,11,12,13]. Pregnant women who are at increased risk of severe COVID-19 including those with advanced age, comorbidities such as obesity, hypertension, chronic lung or heart disease, diabetes, and immunosuppression [14,15], as well as those with high occupational exposure risk such as frontline healthcare workers [16,17]. Despite being clinically vulnerable, pregnant individuals cannot be included in the initial COVID-19 vaccine trials due to obvious safety concerns [18,19]. Thus, the lack of data on the safety and efficacy of these vaccines remained the main contributing factor to the vaccine hesitancy among this population [20] since COVID-19 vaccines rollout in December 2020. 

The term vaccine hesitancy refers to a “delay in accepting or refusing to vaccinate despite the vaccination services being available”. It is among the top ten threats to global health and is influenced by “complacency, convenience, and confidence” [21,22]. The cautious approach usually excludes pregnant women from clinical trials due to safety and legal liability concerns, both for the mother and fetus [23,24]. However, routine antenatal vaccination programmes exist for pertussis, tetanus, diphtheria, polio, and seasonal influenza. In contrast, other vaccines, such as hepatitis vaccine, meningococcal vaccine, and pneumococcal vaccine, are only indicated if there is high exposure risk [25,26]. Pregnant women remain vulnerable to hepatitis E-associated acute liver failure [27]. Furthermore, not all vaccine formulations are safe in pregnancy; for instance, the live measles mumps rubella (MMR) vaccine should be avoided in pregnancy due to potential risk of fetal viremia [28]. 

The inclusion of pregnant women in the COVID-19 vaccine campaign is also confounded by the limited safety data, including the lack of developmental and reproductive toxicology (DART) studies, albeit no reproductive safety concerns were identified in the early discovery data [29,30,31]. Furthermore, similar reactogenicity profiles were observed between groups (pregnant, lactating, and non-pregnant women) receiving mRNA COVID-19 vaccine without associated safety concerns [32]. Data available from other mRNA and adenovirus vector-based vaccine development efforts against influenza, Zika, HIV, rabies, and Ebola viruses are reassuring, with no pregnancy-related adverse events [33,34,35,36,37,38,39,40]. Emerging data collected from the United States’ Centers for Disease Control and Prevention (CDC) registry following mRNA COVID-19 vaccination in pregnant women did not highlight any remarkable safety concerns [41,42]. Recent efforts leading COVID-19 vaccine manufacturers to further evaluate the effects of their COVID-19 vaccines on pregnant women are encouraging [43,44,45], though some rare but severe side effects have been identified during post-marketing surveillance (pharmacovigilance) such as anaphylaxis, immune thrombocytopenia, thrombotic thrombocytopenia and Guillain-Barré syndrome [46,47,48,49,50,51,52,53,54,55]. An observational retrospective study of 927 ongoing pregnancies in Romania that included 124 cases of spontaneous abortions found no significant difference in spontaneous abortion risk in mRNA vaccinated women compared to unvaccinated women during the first trimester of pregnancy [56]. In another observational cohort study in Canada (the Canadian National Vaccine Safety Network Cohort Study, CANVAS) that included 5597 pregnant participants receiving one dose and 3108 receiving two doses, and 339 unvaccinated pregnant participants across seven provinces in Canada, a reassuring safety profile of mRNA vaccine was noted among pregnant women. Pregnant vaccinated females had increased odds of significant health event within seven days of receiving the vaccine after the second dose of mRNA-1273 vaccine (adjusted odds ratio [aOR] 4·4 [95% CI 2·4–8·3]) compared with pregnant unvaccinated controls within the past seven days, but not after the first dose of mRNA-1273 vaccine or any dose of BNT162b2 vaccine [57]. In a systematic review and meta-analysis of nine studies including 40,728 pregnant women (52.3% vaccinated vs. 47.7% unvaccinated), there was no difference in probability of small for gestational age and other adverse perinatal outcomes between vaccinated and unvaccinated pregnant women, who did not acquire COVID-19 during pregnancy. The rate of preterm delivery seems to have reduced in vaccinated pregnant women compared to their unvaccinated counterparts [58]. It should be noted that the COVID-19 vaccine safety data from Romania [56], Canada [57] and the meta-analysis [58] were not published when this study was conducted, and therefore, healthcare professionals and pregnant women who participated in this study had limited information on the safety of COVID-19 during pregnancy.

Recent data released by the United States’ Centers for Disease Control and Prevention (CDC) reported more than 150,036 confirmed cases of COVID-19 among pregnant women, including more than 25,402 hospitalisations and 248 deaths (US) [3]. According to the latest figures from the National Health Service (NHS) in England, pregnant women represent almost 20% of the most critically ill COVID-19 patients [4]. In contrast, a global study conducted across 18 low, middle, and high-income countries, including Pakistan, revealed that pregnant women affected by COVID-19 were 22 times more prone to death and 50% more likely to suffer from pregnancy-related complications [5]. In addition, higher fetal death rates, preterm birth, pre-eclampsia, and emergency caesarean delivery were seen in pregnant women diagnosed with COVID-19 than in their uninfected counterparts [59,60,61,62,63,64,65,66].

Vaccine hesitancy is widespread in many parts of the globe, particularly in low-middle-income countries, due to unchecked, misleading, and false information spreading through social media platforms [67,68,69]. Coupled with limited safety data of COVID-19 vaccine, pregnant women may be more likely to refuse vaccination, which can have serious public health consequences. Therefore, this study aimed to assess vaccination status and factors contributing to vaccine hesitancy among pregnant women in the largest province of Pakistan, using a novel study instrument.

## 2. Methods

### 2.1. Study Design and Population

This is a multicentric, prospective, survey-based study using an interviewer-administered tool conducted among pregnant women attending antenatal clinics (between 1 December 2021 and 30 January 2022) in four district headquarters hospitals (Pakpattan, Okara, Kasur, Vehari) and three tertiary care hospitals (teaching hospitals in Sahiwal, Rawalpindi, and Faisalabad) in Punjab, Pakistan. Pregnant women above 18 years of age, attending antenatal clinics, and providing consent were invited to participate in this study. The survey was administered by the healthcare professionals providing care at participating hospitals. 

### 2.2. The Survey Instrument

The questions included in the study instrument were developed based on the input received from healthcare providers involved in administering COVID-19 vaccination and the potential factors associated with vaccine hesitancy. The initial draft of the study instrument was developed by the investigators (S.S.H. and H.A.M.) that were later shared with four experts: two academicians (one from an epidemiology background and the other with expertise in psychometric testing of questionnaires) and two healthcare professionals (with research experience) to determine the content and face validity of the questionnaire. The experts were given one week to review and provide feedback. A revised version of the study instrument was produced after incorporating changes based on the comments received from reviewers. The revised version was piloted among a small group of the target population to ensure the clarity and feasibility of the survey instrument (see supplementary file).

The final version of the study instrument comprised four sections to gather the required information. The first section of the study instrument (9 items) collected participant-related information (e.g., age, long-standing illness, pregnancy history, gestational week, etc.). The second section (16 items) of the study instrument was about the vaccination status of the participants (e.g., COVID-19 vaccine information, adverse effects following immunisation, vaccination status of family members, etc.). The third (11 items) and fourth (30 items) sections consisted of questions to assess vaccine hesitancy among vaccinated and unvaccinated pregnant women respectively, measured on a five-point Likert scale.

The English version of the study instrument was translated into Urdu using the forward-backward translation method by the study authors who were native Urdu speakers with the help of healthcare professionals involved in the data collection process. This was to maintain consistency among the healthcare professionals involved in the data collection process. All healthcare professionals involved in the data collection possessed knowledge of the health concepts used in the study tool and competently spoke both Urdu and English languages. Conceptual and cultural equivalence was the core of this exercise instead of linguistic equivalence.

### 2.3. Sample and Sampling

The study was approved by the Research Ethics Committee of The University of Lahore (REC/DPP/FOP/39, dated 26 August 2021) and by the District Headquarters Hospital (No. 3714/B/PA/DHQ, dated 15 October 2021). The permission to conduct the study was also obtained from all participating hospitals. We invited all consecutive pregnant women attending antenatal clinics in four district headquarters hospitals and three tertiary care hospitals to participate in the survey. Due to the COVID-19-related restrictions in place in the participating hospitals, an online web-based version of the study tool, with Urdu translation, was developed and used by the healthcare professionals involved in the data collection process. These healthcare professionals accessed the online data collection form through mobile devices. They administered the questionnaire after explaining the study objectives and taking verbal consent from the patients in Urdu, their national language.

### 2.4. Statistical Analysis

Data are presented as frequencies, percentages, mean/median, and standard deviation. The internal consistency or reliability of the study questionnaire was determined using Cronbach’s alpha (α), where the alpha coefficient determines the extent to which multiple indicators belong together for a latent variable. A commonly accepted threshold for reliability is more than or equal to 0.70. However, values below 0.70 are also acceptable. The chi-Squared test was used to compare between the vaccinated and non-vaccinated groups for their demographic characteristics and vaccination-related factors. Independent *t*-test or Mann–Whitney test and one-way ANOVA or Kruskal–Wallis tests were used at a 95% confidence interval (*p* < 0.05) to examine the differences in hesitancy between participants-related factors. Hesitancy scores were calculated as the sum of scale items and transformed into a scale (vaccinated participants: 11 to 55) and (unvaccinated participants: 30 to 150). All scales and component scores were positively scored, with higher scores represent lower hesitancy. Multivariate logistic regression with a backward stepwise model was used to examine the association between vaccination status and hesitancy among participants and vaccine-related factors. The variables were selected in the model based on the *priori* knowledge in the first step: age, education, occupation, long-standing illness, received information about the vaccine, and pregnancy-related problems. The final model was selected based on the model summary with Hosmer & Lemeshow test. All statistical tests were performed using Statistical Package for Social Sciences (SPSS^®^) version 27.

## 3. Results

Out of 650 pregnant women approached to participate, 405 agreed and completed the study (response rate = 62.3%). The study instruments measuring hesitancy in vaccinated (Cronbach’s alpha = 0.896) and unvaccinated (Cronbach’s alpha = 0.897) participants demonstrated good reliability. Overall, the mean age of the study participants was 29.1 years, with most study participants (38.6%, n = 156) aged between 30 and 34 years and 25 and 29 years (32.2%, n = 130), and about half of the study population (56.3%, n = 228) had received either no formal education or were only educated up to primary school level, as shown in Table 1. The majority of the vaccinated and unvaccinated participants were in the third trimester of their pregnancy (46.3%, n = 156 and 53.7%, n = 181). In addition, more unvaccinated study participants had no previous successful pregnancy history (60.6% vs. 39.4%) with higher pregnancy-related issues (80.6% vs. 19.4%) than vaccinated participants.

Table 2 presents the COVID-19 vaccination status, any adverse events following immunisation (AEFI), and future planning to receive COVID-19 vaccines for the study participants. More than half of the study participants (56.0%, n = 227) did not receive full or partial COVID-19 vaccination at the time of the data collection. In contrast, almost all family members of the pregnant women (93.9%, n = 372) had already received at least one dose of the COVID-19 vaccine.

**Figure 1:**
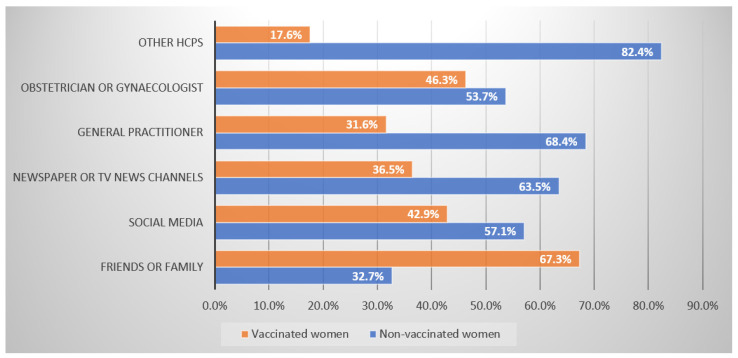
Sources of information about COVID-19 vaccines in vaccinated and non-vaccinated women.

Among the pregnant women who received the COVID-19 vaccine (n = 173), 39.9% (n =69) of them experienced AEFIs. Forty-eight women described their ADR as mild, while 20 experienced moderate AEFIs. Participating women managed their AEFIs mainly by taking over-the-counter products and/or resting at home (n = 46). The majority of the unvaccinated women (77.8%, n = 182) had no planning to receive the vaccine in the future. However, more than two-thirds (85.7%, n = 342) consulted the doctor about COVID-19 vaccines, and most were recommended to receive COVID-19 vaccines by their doctors (80.7%, n = 280). Unvaccinated women stated other HCPs (82.4%), general practitioners (68.4%), and social media (57.1%) as the main sources of information about COVID-19 vaccines. On the other hand, vaccinated women stated friends or family (67.3%) and obstetricians and gynaecologists (46.3%) as the most frequently used sources of information about COVID-19 vaccines (Figure 1).

Occupation, long-standing illness, consultation with the doctor, and pregnancy-related problems were significant variables in multivariate logistic analysis of variables affecting COVID-19 vaccination, with women being significantly more likely to be vaccinated if they had employment (OR 4.47, 95% CI: 2.31–8.64) compared with homemakers, consulted their doctors (OR 0.12, 95% CI: 0.04–0.35) and if they did not have the pregnancy-related issue (OR 6.02, 95% CI: 2.36–15.33). There was no significant difference in COVID-19 vaccination status based on trimesters (Table 3).

Variable(s) entered on step 1: Age, Education, Occupation, Trimesters, any pregnancy issue, long-standing illness, received information during pregnancy, family members fully or partially vaccinated, consult your doctor. CI = confidence interval. An odds ratio > 1 represents higher odds of being vaccinated.

Among pregnant women who had received COVID-19 vaccines, 44.3% (n = 79) indicated that they had taken the vaccines because of their efficacy in protecting them against COVID-19, 40.4 % (n = 72) and 43.2% (n = 77) considered COVID-19 vaccines were important for themselves and for their babies, respectively. Most of the participating women stated that COVID-19 vaccines would reduce the chance of COVID-associated hospitalisation (42.1%, n = 75), were as safe as other vaccines (40.4%, n = 72), and provided more benefits in pregnancy than the risks (38.7%, n = 69), as shown in Table 4. More than half of the study population (56.1%, n = 100) trusted the vaccine information provided by the government authorities on COVID-19 vaccine safety, while 15.7% (n = 28) did not trust the authorities. 

**Figure 2:**
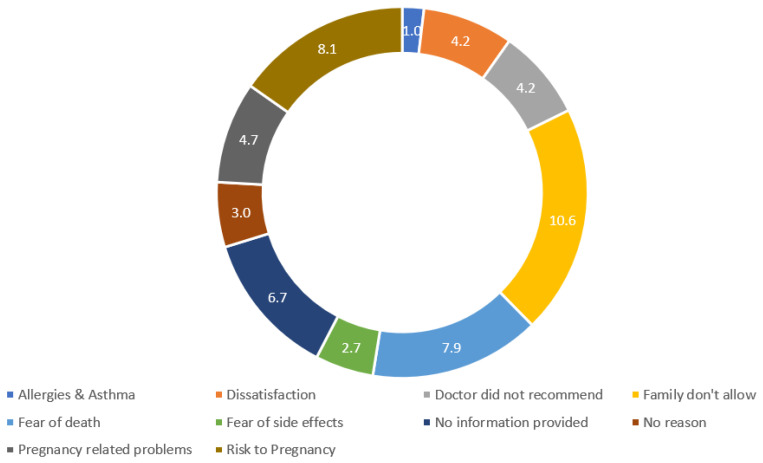
Reasons for not having vaccines in percentage (n = 227).

The most common reasons for not receiving COVID-19 vaccines among unvaccinated participants in this study included family pressure (10.6%), fear of death (7.9%), allergies and asthma (4.7%), and lack of information (6.7%), as summarised in Figure 2.

Participating women who did not receive COVID-19 vaccines considered COVID-19 vaccine: not important for the health of their babies (42.2%, n = 96); not effective in lowering the risk of getting COVID-19 (37.8%, n = 86); or lowering the risk of hospitalization due to COVID-19 (40.9%, n = 93); and lack trust on the information provided by the health authorities about the safety of COVID-19 vaccines in pregnancy (40%, n = 91) as shown in Table 5. Astonishingly, about half of the participating women (49.7%, n = 113) agreed that they would die in the next two years if they receive COVID-19 vaccines. Furthermore, over half of the participating women did not have the independence on immunisation decisions for themselves and reported pressures from their spouse or close family not to receive COVID-19 vaccines (51.5%, n = 117). Surprisingly, around two-thirds of pregnant women (65.1%, n = 148) would prefer spiritual treatment over COVID-19 vaccines.

Table S1 (in supplementary files) summarised the descriptive statistics for the vaccine hesitance scales. The median hesitancy scores for vaccinated and unvaccinated women were 28.0 (IQR: 26–32) and 82.0 (IQR: 76–91). The total actual scores ranged from 51 to 121 (possible score range: 30 to 150) in unvaccinated women. Table S2 presents the mean hesitancy score according to age groups, education level, pregnancy-related factors, and COVID-19-related information. Overall, no significant differences in vaccine hesitancy were observed according to age, presence of chronic illness, and presence of pregnancy-related issues. However, in pair-wise comparisons, women who were employed had significantly lower hesitancy than women who were housemakers (mean score 90.2 vs. 81.8, *p* = 0.001). In a multivariate linear regression model, only occupation was significantly associated with hesitancy score (coefficient −4.32, 95% CI: −4.54, −1.69, *p* = 0.001). 

## 4. Discussion

COVID-19 vaccine hesitancy among pregnant subjects has been a global concern. In the United States, a cross-sectional, survey-based study which was undertaken at an academic medical centre to understand the COVID-19 vaccine hesitancy among reproductive-aged female healthcare workers reported that pregnant women or those trying to conceive (TTC) significantly more likely to refuse or defer COVID-19 vaccination [70]. The pregnant population had six times the odds of deferring the vaccination and was twice as likely to refuse the vaccination than other female participants of reproductive age. In contrast, TTC were nearly three times more likely to defer or refuse the vaccination than other female participants of reproductive age [70]. In another cross-sectional survey which was conducted in the United States to evaluate pregnant women’s attitudes toward COVID-19 vaccination and factors associated with their acceptance, only 41% of participants were willing to get the COVID-19 vaccine if offered. The most common concern for refusal was vaccine safety (82%), while those with prior influenza vaccine history were more prone to vaccination [71]. Finally, insufficient research and concerns of potential foetal harm were reported as the two major reasons for the low vaccine acceptance rate (44%) among pregnant subjects in another US medical center. Additionally, the same respondents had the highest rate of vaccine refusal (27%) [72]. 

During the first wave of the COVID-19 pandemic, pregnant women’s willingness to vaccinate and its associated factors were explored in a multinational, cross-sectional study through the distribution of an online survey across six European countries, including Belgium, Ireland, Norway, Netherlands, Switzerland, and the United Kingdom (UK). Respondents with low levels of education and unemployment were less likely to receive COVID-19 vaccination [72]. To evaluate the level of COVID-19 vaccine acceptance among pregnant women as well as their potential predictors, a cross-sectional online survey was conducted among sixteen countries, including Australia, Argentina, Brazil, Colombia, Chile, Italy, India, Mexico, New Zealand, Peru, Philippines, Russia, Spain, South Africa, US, and the UK. About 52% of pregnant respondents intended to be vaccinated, assuming that the vaccine would be 90% effective. Feeling confident about the safety and efficacy of the COVID-19 vaccine and routine vaccines, recognising the value of vaccination, being concerned about COVID-19, complying with COVID-19 guidelines, and trusting the public health system were among the key predictors associated with COVID-19 vaccine acceptance [73].

The unprecedented pace at which the COVID-19 vaccines were developed and accessed has also contributed to vaccine hesitancy among pregnant individuals [74]. Traditionally, vaccine development was a complex and time-consuming process with a typical timeline of 10–15 years that included extensive studies at each stage of discovery and development, including lead identification, early toxicology and immunology screening, clinical evaluation (phase I-III), scale-up, and large-scale production, quality, and regulatory dossiers. The vaccine ultimately hits the market, followed by a long and extensive post-marketing surveillance, and pharmacovigilance, aka phase IV studies [75]. On the contrary, the COVID-19 vaccines was developed remarkably within 12–24 months by building on platform technologies that had been previously exploited for other vaccines, open-source knowledge about virus morphology and potential antigenic sites, open-source gene sequencing, combined phase I and II trials, parallel efforts in formulation scale-up to facilitate mass production of the vaccine, expedited regulatory review process [76].

Healthcare professionals, in particular those involved in providing prenatal care, play a significant role in addressing vaccine hesitancy in pregnant populations. According to a French online survey, most healthcare professionals favored vaccinating pregnant individuals; however, some had reservations and were less likely to make recommendations on COVID-19 vaccination. In addition, there was a positive association between agreement with COVID-19 vaccination among pregnant women and those who were obstetricians, worked in a group, and usually offered other vaccines [77]. In order to assess the COVID-19 vaccine willingness among pregnant women in Switzerland, a cross-sectional online study was undertaken. Had the vaccine been available against COVID-19 during the first pandemic, only 29.7% of pregnant women showed willingness to get the vaccine. The strongest predictors associated with COVID-19 vaccine acceptance included advanced maternal age and level of education, prior influenza vaccination status, having an obstetrician involved in their care, and being in the third trimester [78]. A multicentre, cross-sectional, online survey-based study was also conducted in Italy to evaluate the perspectives of pregnant women on COVID-19 vaccination. Most pregnant respondents (71.4%) were not in favour of getting vaccinated, and their pregnancy status guided the ultimate choice. Concerns of potential consequences on baby’s health and the lack of safety data associated with producing these vaccines were the most common reasons for the refusal [79]. 

In an attempt to investigate the predictors of the intention of pregnant women toward COVID-19 vaccination, a cross-sectional study involving face-to-face interviews was carried out in Bench-Sheko Zone, Southwest Ethiopia, among pregnant women who attended antenatal care services at selected public health facilities. About 31% of the study participants intended to receive COVID-19 vaccination once the vaccines became available [80]. This finding was much lower than the study conducted in Gurage Zone, Southwest Ethiopia, where the COVID-19 vaccine acceptance among pregnant women was 70.9% [81]. However, advanced maternal age, higher educational backgrounds, urban residence, compliance with government COVID-19 guidelines, and positive perception of the COVID-19 vaccines were among the most important predictors of participants’ intention to be vaccinated against COVID-19 in these studies.

Another prospective study utilising a face-to-face questionnaire was undertaken to assess COVID-19 vaccine acceptance and hesitancy among pregnant women attending antenatal care services in Ankara, Turkey. About 37% participants had positive intention to receive COVID-19 vaccination if official recommendations were made for this population. The lack of vaccine safety data in pregnant women in the study was the greatest concern and major contributing factor to vaccine refusal. Women in their first trimester expressed higher vaccine acceptance than participants in their second and third trimesters [82]. About 47% of pregnant women in Turkey did not intend to be vaccinated against COVID-19; a higher proportion than the 29.6% of this population showed hesitancy towards vaccinations [83]. The situation in the Middle East was similar, where an online-based cross-sectional survey which was conducted among pregnant women in Qatar, exhibiting a COVID-19 vaccine hesitancy rate of 25%, with vaccine safety being the main factor identified [84]. A cross-sectional study recruiting 184 pregnant women from obstetrics and gynecology clinics in Romania had significantly higher vaccine hesitancy scores than non-pregnant women. The determinants of hesitancy recorded were ‘being not afraid of COVID-19′, ‘low income’, ‘social media’, ‘not believing the virus existence’, and ‘vaccine non-believer’ [85]. In another qualitative study that involved 92 respondents from 10 provinces with different gross domestic product(GDP) levels in China were interviewed using a semi-structured face-to-face interview, the major contributors for vaccine hesitancy identified were concerns over safety and access to professional advice, followed by vaccine price and affordability and perceived poor efficacy [86]. Recently, COVID-19 vaccine acceptance was also explored in countries like Saudi Arabia in a cross-sectional study using an online questionnaire where pregnant women and those planning pregnancy were more hesitant toward COVID-19 vaccination than others [87].

There are limitations associated with this study, including that the data was obtained only in Punjab, which limited the generalisability of our findings since vaccination uptake probably varies greatly within the country. There was also a risk of selection bias, since those attending the antenatal clinics were being more likely to be included as participants. Furthermore, this study did not investigate potential factors that could affect vaccine uptakes.

## 5. Conclusions

The study found a high COVID-19 vaccine hesitancy rate among pregnant women in Pakistan. More than half of the study participants remained unvaccinated at the time of the study, even though most of their family members hadalready vaccinated. The majority of the unvaccinated women had no intention to get the vaccine, and a significant number of women relied on social media to seek information on vaccine. However, some women reported no trust in the information provided by the health authorities. Family pressure, fear of death, and allergies were the most common reasons for refusing COVID vaccines. It was also noted that the educated or employed women were less hesitant to receive COVID vaccines than those without formal education or housemakers. 

## Figures and Tables

**Table 1:** Demographics and pregnancy-related factors.

Items	Non-Vaccinated Women n (%)	Vaccinated Women n (%)	*p*-Value
Age, mean SD	28.8 (4.5)	29.5 (4.2)	0.098
Age categories (n = 404)			
*18–24*	43 (66.2)	22 (33.8)	0.084
*25–29*	75 (57.7)	55 (42.3)	
*30–34*	76 (48.7)	80 (51.3)	
*35–39*	32 (60.4)	21 (39.6)	
Education (n = 405)			
*No formal education*	58 (69.9)	25 (30.1)	0.002
*Primary education*	81 (55.9)	64 (44.1)	
*Secondary education*	51 (60.7)	33 (39.3)	
*Diploma*	24 (41.4)	34 (58.6)	
*Graduation and above*	13 (37.1)	22 (62.9)	
Occupation (n = 405)			
*Employed*	29 (33.0)	59 (67.0)	0.001
*Unemployed*	18 (66.7)	9 (33.3)	
*Self-employed*	39 (54.2)	33 (45.8)	
*Homemaker*	141 (64.7)	77 (35.3)	
Long-standing illness			
*No*	189 (52.1)	174 (47.9)	0.001
*Yes*	26 (96.3)	1 (3.7)	
Gestational week, mean SD (n = 405)	30.3 (6.7)	32.2 (5.9)	0.003
*First Trimester*	5 (50.0)	5 (50.0)	0.046
*Second Trimester*	41 (70.7)	17 (29.3)	
*Third Trimester*	181 (53.7)	156 (46.3)	
Previous successful pregnancy history (n = 404)			
*No*	57 (60.6)	37 (39.4)	0.321
*Yes*	170 (54.8)	140 (45.2)	
Any pregnancy-related issue (n = 396)			
*No*	190 (52.8)	170 (47.2)	0.001
*Yes*	29 (80.6)	7 (19.4)	

**Table 2:** Vaccination status and participants’ experiences with the COVID-19 vaccine.

Items	n (%)
Received full or partial COVID-19 vaccination (n = 405)	178 (44.0)
Family members received full or partial vaccination (n = 396)	372 (93.9)
If you have only taken one dose of the vaccine, why? (n = 105)	
*Experienced AEFIs*	25 (23.8)
*One dose was enough for me*	2 (1.9)
*Caught COVID-19 or was not well and missed the second dose*	4 (3.8)
*Other*	74 (70.5)
Take annual or booster dose (n = 167)	121 (72.5)
Experienced any adverse reactions following the COVID-19 vaccine (n = 173)	69 (39.9)
Describe AEFI severity (n = 71)	
*Mild*	48 (67.6)
*Moderate*	20 (28.2)
*Severity*	3 (4.2)
AEFIs and vaccine doses (n = 68)	
*First dose*	54 (79.4)
*Second dose*	7 (10.3)
*Both*	7 (10.3)
How did you manage your AEFIs (n = 67)	
*Consulted a medical doctor*	3 (4.5)
*Took OTC products and rested at home*	46 (68.7)
*Admitted to a hospital*	18 (26.9)
Planning to receive COVID-19 vaccine in the future (n = 234)	52 (22.2)
Received information about vaccine during pregnancy (n = 400)	367 (91.8)
If you were concerned about vaccine safety during pregnancy, have you tried to consult your doctor for advice? (n = 399)	342 (85.7)
If yes, did they recommend taking the vaccine during pregnancy? (n = 347)	280 (80.7)
Overall, are you satisfied with the COVID-19 vaccine? (n = 175)	137 (78.3)

**Table 3:** Variables affecting COVID-19 vaccination based on multivariate analysis.

Items	No. of Participants	OR (95% CI)	*p*-Value
Occupation			
*Employed*	29	4.47 (2.31–8.64)	0.001
*Unemployed*	18	2.10 (0.68–6.35)	0.199
*Self-employed*	39	2.37 (1.19–4.69)	0.013
*Housemaker*	141	Reference	
Consulted your doctor			
*No*	57	0.12 (0.04–0.35)	0.001
*Yes*	342	Reference	
Any pregnancy-related issue			
*No*	190	6.02 (2.36–15.33)	0.001
*Yes*	29	Reference	
Received COVID-19-related information			
*No*	30	0.28 (0.07–1.16)	0.079
*Yes*	196	Reference	

**Table 4:** Responses to items measuring hesitancy among vaccinated pregnant women (n = 178).

I Have Taken the COVID-19 Vaccine Because	Agreement n (%)	Disagreement n (%)	Mean	SD
the vaccine is an effective way of protecting pregnant women from COVID-19 infection	79 (44.3)	20 (11.2)	2.67	0.68
the vaccine is important for my health during pregnancy	72 (40.4)	20 (11.2)	2.71	0.68
the vaccine is important for the health of my baby (foetus)	77 (43.2)	19 (10.6)	2.69	0.69
vaccine lowers the risk of COVID-19 infection during pregnancy	80 (44.9)	22 (12.3)	2.66	0.73
vaccines prevent COVID-19 infection from becoming worse and the need for hospitalization	75 (42.1)	20 (11.2)	2.69	0.67
vaccine is a more effective preventive measure than using natural or other remedies	78 (43.8)	22 (12.3)	2.69	0.71
COVID-19 vaccines are as safe as other vaccines that are normally used during pregnancy (e.g., flu)	72 (40.4)	23 (12.9)	2.73	0.74
the benefits of the COVID-19 vaccine during pregnancy outweigh its risks	69 (38.7)	19 (10.6)	2.73	0.71
there are not many adverse effects reported for COVID-19 vaccines	62 (34.8)	28 (15.7)	2.85	0.80
sufficient information is available about the long-term safety and efficacy of COVID-19	71 (39.8)	21 (11.7)	2.72	0.69
I trust information shared by government or public health agencies about the efficacy and safety profile of COVID-19 vaccines	100 (56.1)	28 (15.7)	2.64	0.84

Strongly agree or agree = agreement and strongly disagree or disagree = disagreement.

**Table 5:** Responses to items measuring hesitancy among unvaccinated pregnant women (n = 227).

I Have Not Taken the COVID-19 Vaccine Because	Agreement n (%)	Disagreement n (%)	Mean	SD
the vaccine is not an effective way of protecting pregnant women from COVID-19 infection	44 (19.3)	32 (14.0)	2.94	0.64
the vaccine is not important for my health during pregnancy	72 (31.7)	33 (14.5)	2.81	0.71
the vaccine is not important for the health of my baby (foetus)	96 (42.2)	27 (11.8)	2.68	0.72
vaccine does not lower the risk of COVID infection during pregnancy	86 (37.8)	27 (11.8)	2.73	0.73
vaccine does not prevent COVID infection from becoming worse, and the need for hospitalization	93 (40.9)	32 (14.0)	2.73	0.78
using natural or other remedies are more effective than COVID vaccines	78 (34.3)	34 (14.9)	2.78	0.76
using natural or other remedies are safer than COVID vaccines	78 (34.3)	25 (11.0)	2.74	0.74
COVID vaccines are not as safe as other vaccines that are normally used during pregnancy (e.g., flu)	75 (33.0)	30 (13.2)	2.81	0.73
the benefits of the COVID vaccine during pregnancy do not outweigh the risks	74 (32.5)	22 (9.6)	2.75	0.69
there are too many adverse effects reported for COVID vaccines	74 (32.5)	29 (12.7)	2.77	0.70
insufficient information is available about the long-term safety and efficacy of COVID vaccines	74 (32.5)	27 (11.8)	2.79	0.69
I do not trust information shared by government or public health agencies about the efficacy and safety profile of COVID vaccines	91 (40.0)	26 (11.4)	2.70	0.70
COVID is a conspiracy, and I am not worried about catching COVID infection during my pregnancy	73 (32.1)	25 (11.0)	2.77	0.69
COVID is real, but I think it won’t do any harm to me during pregnancy	67 (29.5)	29 (12.7)	2.84	0.73
COVID is real, but I think it won’t do any harm to my baby during pregnancy	70 (30.8)	31 (13.6)	2.83	0.74
I am concerned that the vaccine will have more harmful effects on my baby than COVID-19 itself	78 (34.3)	29 (12.7)	2.76	0.71
COVID vaccines available in my country are not as effective as those in Western countries	65 (28.6)	27 (11.8)	2.82	0.70
COVID vaccines available in my country are not as safe as those in Western countries	41 (18.0)	35 (15.4)	2.97	0.64
COVID vaccines available in my country are not fit for travel abroad	65 (28.6)	33 (14.5)	2.85	0.71
COVID vaccines are not Halal and are prohibited by my religious beliefs	54 (23.7)	30 (13.2)	2.89	0.66
COVID vaccines contain animal ingredients, and I don’t take medications containing animal ingredients	53 (23.3)	53 (23.3)	3.01	0.74
COVID vaccines will adversely affect my ability to become pregnant or have babies in future	65 (28.6)	34 (14.9)	2.84	0.72
I will die in two years if I take the COVID-19 vaccines	113 (49.7)	17 (7.4)	2.53	0.74
my husband or other family members will not allow me to take the COVID vaccine	117 (51.5)	14 (6.1)	2.48	0.73
my doctor has not encouraged me to take the COVID-19 vaccine during pregnancy	68 (29.9)	49 (21.5)	2.91	0.81
my religious scholars have advised me not to take the COVID-19 vaccine	62 (27.3)	21 (9.2)	2.81	0.66
I have not taken the vaccine because the vaccine brand that I wanted was not available in my region	54 (23.7)	35 (15.4)	2.92	0.67
I usually do not trust or believe in vaccines and do not take vaccines in general	59 (25.9)	26 (11.4)	2.86	0.67
herbal or natural treatment is better than COVID vaccine	63 (27.7)	30 (13.2)	2.82	0.71
spiritual treatment is better than taking vaccines	148 (65.1)	17 (7.4)	2.34	0.76

Strongly agree or agree = agreement and strongly disagree or disagree = disagreement.

## Data Availability

The datasets generated during and/or analyzed during the current study are available from the corresponding author upon reasonable request.

## Supplementary Materials

The following supporting information can be downloaded at: https://www.mdpi.com/article/10.3390/v14112344/s1, Table S1: Descriptive statistics for the vaccine hesitancy scales in vaccinated and unvaccinated women. Table S2: Vaccine hesitancy among unvaccinated pregnant women by participants-related factors.
