# Supplementary material for: COVID-19 Vaccine Hesitancy among Pregnant Women Attending Antenatal Clinics in Pakistan: A Multicentric, Prospective, Survey-Based Study
Source: Viruses. 2022 Oct 25;14(11):2344. doi: 10.3390/v14112344 (PMC9694328; doi:10.3390/v14112344)
Supplement: Supplementary file 1 [file viruses-14-02344-s001.zip › viruses-1943474-supplementary.pdf]

Table S1: Descriptive statistics for the vaccine hesitancy scales in vaccinated and unvaccinated women

Item	Number of items	Scale	Possible range	Actual range	Mean	SD	Median	IQR	Skewness
**Hesitancy scale for vaccinated women**	11	1-5	11-55	17-46	29.7	5.5	28.0	26-32	1.12
**Hesitancy scale for unvaccinated women**	30	1-5	30-150	51-121	83.8	10.8	82.0	76-91	0.45

Note: Higher score in vaccine hesitancy scale for vaccinated women indicates higher hesitancy, whereas higher score in vaccine hesitancy score for unvaccinated women indicates lower hesitancy

Table S2: Vaccine hesitancy among unvaccinated pregnant women by participants-related factors

Items	No. of participants	Hesitancy Score Mean (SD)	*p*-value
Age categories			
18-24	43	81.9 (9.6)	0.554
25-29	75	83.7 (12.1)	
30-34	76	85.1 (9.3)	
35-39	32	84.3 (10.6)	
Education			
No formal education	58	81.2 (10.8)	0.094
Primary education	81	83.2 (9.4)	
Secondary education	51	84.8 (12.9)	
Diploma	24	87.6 (9.6)	
Graduation and above	13	88.0 (10.1)	
Occupation			
Employed	29	90.2 (10.2)	0.001
Unemployed	18	88.1 (13.6)	
Self-employed	39	84.8 (8.9)	
Housemaker	141	81.8 (10.4)	
Long-standing illness			
No	189	83.5 (10.9)	0.752
Yes	26	84.2 (8.8)	
Gestational week			
First Trimester	5	90.0 (3.5)	0.083
Second Trimester	41	80.8 (12.7)	
Third Trimester	181	84.4 (10.2)	

Any pregnancy-related issue			
No	190	83.7 (10.9)	0.417
Yes	29	85.5 (10.4)	
Received COVID-19 related information			
No	30	85.3 (13.1)	0.422
Yes	196	83.5 (10.3)	

Items measuring vaccine hesitancy in vaccinated and unvaccinated women. All items were measured on a 5-point Likert scale

**I have taken the vaccine because (for vaccinated women)**	**I have not taken the vaccine because (for unvaccinated women)**
the vaccine is an effective way of protecting pregnant women from COVID-19 infection	vaccine does not lower the risk of COVID infection during pregnancy (Q2c)
the vaccine is important for my health during pregnancy	using natural or other remedies are more effective than COVID vaccines (Q2e)
the vaccine is important for the health of my baby (foetus)	COVID vaccines are not Halal and are prohibited by my religious beliefs
vaccine lowers the risk of COVID-19 infection during pregnancy	COVID vaccines will adversely affect my ability to become pregnant or have babies in future
vaccines prevent COVID-19 infection from becoming worse and the need for hospitalization	I am concerned that the vaccine will have more harmful effects on my baby than COVID-19 itself
vaccine is a more effective preventive measure than using natural or other remedies	my religious scholars have advised me not to take the COVID-19 vaccine (Q2z)
COVID-19 vaccines are as safe as other vaccines that are normally used during pregnancy (e.g., flu)	herbal or natural treatment is better than COVID vaccine (Q2cc)
the benefits of the COVID-19 vaccine during pregnancy outweigh its risks	the vaccine is not an effective way of protecting pregnant women from COVID-19 infection (Q2a)
there are not many adverse effects reported for COVID-19 vaccines	the vaccine is not important for the health of my baby (foetus) (Q2c)
sufficient information is available about the long-term safety and efficacy of COVID-19	vaccine does not prevent COVID infection from becoming worse, and the need for hospitalization (Q2d)
I trust information shared by government or public health agencies about the efficacy and safety profile of COVID-19 vaccines	using natural or other remedies are safer than COVID vaccines
	the benefits of the COVID vaccine during pregnancy do not outweigh the risks
	insufficient information is available about the long-term safety and efficacy of COVID vaccines
	COVID is a conspiracy, and I am not worried about catching COVID infection during my pregnancy

	my doctor has not encouraged me to take the COVID-19 vaccine during pregnancy (Q2y)
	the vaccine is not important for my health during pregnancy (Q2b)
	there are too many adverse effects reported for COVID vaccines
	I do not trust information shared by government or public health agencies about the efficacy and safety profile of COVID vaccines
	my husband or other family members will not allow me to take the COVID vaccine
	spiritual treatment is better than taking vaccines (Q2dd)
	COVID vaccines are not as safe as other vaccines that are normally used during pregnancy (e.g., flu)
	COVID vaccines available in my country are not as effective as those in Western countries
	COVID vaccines available in my country are not as safe as those in Western countries
	COVID vaccines available in my country are not fit for travel abroad
	COVID vaccines contain animal ingredients, and I don't take medications containing animal ingredients
	COVID is real, but I think it won't do any harm to me during pregnancy
	COVID is real, but I think it won't do any harm to my baby during pregnancy
	I will die in two years if I take the COVID-19 vaccines
	I have not taken the vaccine because the vaccine brand that I wanted was not available in my region (Q2aa)
	I usually do not trust or believe in vaccines and do not take vaccines in general (Q2bb)
